# Sparsely sampled MR navigators as a practical tool for quality control and correction of head motion in simultaneous PET/MR

**DOI:** 10.1186/2197-7364-1-S1-A36

**Published:** 2014-07-29

**Authors:** Sune H Keller, Casper Hansen, Christian Hansen, Flemming L Andersen, Claes Ladefoged, Claus Svarer, Andreas Kjær, Liselotte Højgaard, Ian Law, Otto M Henriksen, Adam E Hansen

**Affiliations:** Dept. of Clinical Physiology, Nuclear Medicine and PET, Rigshospitalet (Copenhagen University Hospital), Kragujevac, Denmark; Neurobiology Research Unit, Rigshospitalet (Copenhagen University Hospital), Kragujevac, Denmark

We present a study using MR navigators sampled between other protocolled MR sequences during PET/MR brain scanning, to make motion-QC and motion correction (MC) feasible in clinical simultaneous PET/MR, obviating the need for special MR sequences with interleaved (dense) navigators.

During 30 min [11C]-PiB (453±148 MBq) PET listmode scans of 29 patients on the Siemens mMR, a set of 10 3D navigator volumes was acquired (2D EPI 3.0x3.0x3.0 mm^3^ voxels, 64x64 matrix, 36 slices, TE 30 ms, TR 3,000 ms) in between other scheduled MR acquisitions at 6 time points. The rigid motion between navigators was calculated (all motions given are for a point in the cortex 6 cm from the scanners cFOV).

The 3 patients with largest motion (11, 8 and 8 mm) and the only 2 PiB-positive patients with motion > 4 mm (8 and 6 mm motion) were reconstructed into 6 frames with frames being split at the midpoint between navigator start times (framing [mean±SD]: 259±8, 306±8, 191±2, 342±2, 546±1, 157±19 seconds) and then averaged to one image after MC. A blinded evaluation comparing non-MC and MC PiB images was performed by a nuclear medicine physician.

The average maximum motion magnitude was 3.9±2.4 mm (1 to 11 mm). The visual evaluation of the 5 MC patients rated 3/5 non-MC images blurred (average score: 3.4±0.5 on a scale of 1-5) and 0/5 MC images blurred (score: 4.0±0.0) with no correlation between motion magnitude and rating.

A slight, overall decrease in blurring and an increase in image quality rating with MC was found, but with no impact on clinical outcome. The effect of MC is limited with PiB, but with other tracers (e.g. FDG), longer scans, smaller ROIs or larger motion magnitudes, navigators are easily used for motion-QC and MC in clinical practice.

